# Low-Copy Number Polymorphism in DEFA1/DEFA3 Is Associated with Susceptibility to Hospital-Acquired Infections in Critically Ill Patients

**DOI:** 10.1155/2018/2152650

**Published:** 2018-05-22

**Authors:** Jialian Zhao, Qiang Gu, Lifeng Wang, Weize Xu, Lihua Chu, Ya Wang, Zhongwang Li, Shuijing Wu, Jianguo Xu, Zhiyong Hu, Qiang Shu, Xiangming Fang

**Affiliations:** ^1^Departments of Anesthesiology, the Children's Hospital, School of Medicine, Zhejiang University, Hangzhou, Zhejiang, China; ^2^Departments of Anesthesiology and Intensive Care Unit, First Affiliated Hospital, School of Medicine, Zhejiang University, Zhejiang, Hangzhou, China; ^3^The Children's Hospital, School of Medicine, Zhejiang University, Zhejiang, Hangzhou, China

## Abstract

DEFA1/DEFA3, genes encoding human neutrophil peptides (HNP) 1–3, display wide-ranging copy number variations (CNVs) and is functionally associated with innate immunity and infections. To identify potential associations between DEFA1/DEFA3 CNV and hospital-acquired infections (HAIs), we enrolled 106 patients with HAIs and 109 controls in the intensive care unit (ICU) and examined their DEFA1/DEFA3 CNVs. DEFA1/DEFA3 copy number ranged from 2 to 16 per diploid genome in all 215 critically ill patients, with a median of 7 copies. In HAIs, DEFA1/DEFA3 CNV varied from 2 to 12 with a median of 6, which was significantly lower than that in controls (2 to 16 with a median of 8, *p* = 0.017). Patients with lower DEFA1/DEFA3 copy number (CNV < 7) were far more common in HAIs than in controls (52.8% in HAIs versus 35.8% in controls; *p* = 0.014; OR, 2.010; 95% CI, 1.164–3.472). The area under the receiver operating characteristic (AUROC) of DEFA1/DEFA3 CNV combined with clinical characteristics to predict the incidence of HAIs was 0.763 (95% CI 0.700–0.827), showing strong predictive ability. Therefore, lower DEFA1/DEFA3 copy number contributes to higher susceptibility to HAIs in critically ill patients, and DEFA1/DEFA3 CNV is a significant hereditary factor for predicting HAIs.

## 1. Introduction

Hospital-acquired infections (HAIs) are considered one of the most frequent adverse events that threaten patients' safety in healthcare settings throughout the world [1–3]. Critically ill patients in the intensive care unit (ICU) are extremely vulnerable to HAIs due to their severe illness status, malnutrition, increased invasive treatments, impaired immune function, and even the contaminated environment [3–5]. Currently, HAIs have become a serious issue in ICUs due to their high morbidity (nearly 15.1–47.9% all over the world), high mortality (27.6% or even higher), prolonged length of hospital stay, and increased economic burden [6], but there are no efficient tools for predicting HAIs. Studies show that among critically ill patients, the immune system undergoes simultaneous activation and suppression, leading to severe and persistent immune dysregulation [7, 8], and ultimately, immune suppression may contribute to increased risk for HAIs.

Defensins are small cationic, amphiphilic, cysteine-rich peptides produced by certain leukocytes and epithelial cells and comprise the front line of innate immune defense against pathogens [9–11]. Among them, *α*-defensins 1–3, also called human neutrophil peptides 1–3 (HNP1–3), are mainly expressed in neutrophils with antimicrobial activity against gram-negative and gram-positive bacteria, fungi, and viruses [12, 13]. Besides directly killing pathogens, HNP1–3 also facilitate the activation of adaptive immunity through chemoattracting naive T cells, immature dendritic cells, and monocytes to sites of inflammation [11, 14]. Therefore, HNP1–3 play an important role in the occurrence and development of infections.

DEFA1 and DEFA3, genes that encode HNP1–3, differ by only a single nonsynonymous coding variant within the third exon and are arrayed in a tandem but slightly random pattern of repeats on chromosome 8p23.1, displaying wide-ranging copy number variations (CNVs) [15, 16]. CNVs are defined as deletions, insertions, or duplications of a region of DNA 1 kilobase or larger in length and have significant impacts on genetic variation in the human genome by altering gene dosage, disrupting coding sequences, or perturbing long-range gene regulation [17]. DEFA1/DEFA3 copy number is reportedly correlated with protein levels of HNP1–3 in neutrophils and other expressive sites. In addition, DEFA1/DEFA3 CNV has been associated with inflammation, infection, and several autoimmune diseases [17–20]. However, whether DEFA1/DEFA3 CNV results in individual differences of hereditary susceptibility to HAIs remains uncertain.

In order to verify the association between DEFA1/DEFA3 CNV and HAIs in critically ill patients, we conducted a case-control study of two independent critically ill cohorts of Chinese Han people. We enrolled cases with HAIs and controls without HAIs from three ICUs of the First Affiliated Hospital of Zhejiang University, measured their DEFA1/DEFA3 CNV, and analyzed the function of DEFA1/DEFA3 CNV in the pathogenesis of HAIs.

## 2. Materials and Methods

### 2.1. Study Design, Setting, and Patient Identification

The Ethics Committee (2016450) at the First Affiliated Hospital of Zhejiang University (Hangzhou, China) approved this study. Written informed consent was obtained from the patient or authorized legal representative. We enrolled critically ill patients with HAIs and controls from the surgical ICU, general ICU, and emergency ICU. Infection was defined as hospital acquired when it originated in the hospital environment and appeared 48 hours or more following admission [21], which was diagnosed based on the US Centers for Disease Control and Prevention (CDC) and National Healthcare Safety Network (NHSN) criteria [22]. Exclusion criteria included ages younger than 18 years old, an ICU stay of less than 48 h, immune deficiency including acquired immune deficiency syndrome (AIDS), treatment with corticosteroids, chemotherapy, radiation therapy within 4 weeks, or a history of bone marrow or liver transplantation.

Acute Physiology Score (APS), Acute Physiology and Chronic Health Evaluation score II (APACHE II) [23], and Sequential Organ Failure Assessment (SOFA) [24] scores were obtained in the first 24 h after ICU admission. Diagnosis, any invasive procedures performed within 24 hours, infection sites, culture of pathogens, drug resistance, ICU stays, hospital stays, and 28-day survival were also obtained for further analysis. Multiple infection refers to infection caused by at least two pathogens. Multidrug-resistant bacteria are defined as isolated pathogens with resistance to at least three types of antimicrobial drugs [25].

### 2.2. Samples and DNA Extraction

DEFA1/DEFA3 CNV was determined by quantitative polymerase chain reaction (qPCR) using peripheral whole blood samples. One milliliter of EDTA-stabilized peripheral whole blood sample was obtained from patients within 24 h after diagnosis of HAIs and controls. Genomic DNA was isolated from whole blood samples using the QIAamp DNA Blood Mini Kit (Qiagen, Hilden, Germany) according to the manufacturer's instructions. DNA concentration was measured by spectrophotometry, and samples were stored at −80°C for further analysis.

### 2.3. CNV Determination by qPCR

**Figure 1:**
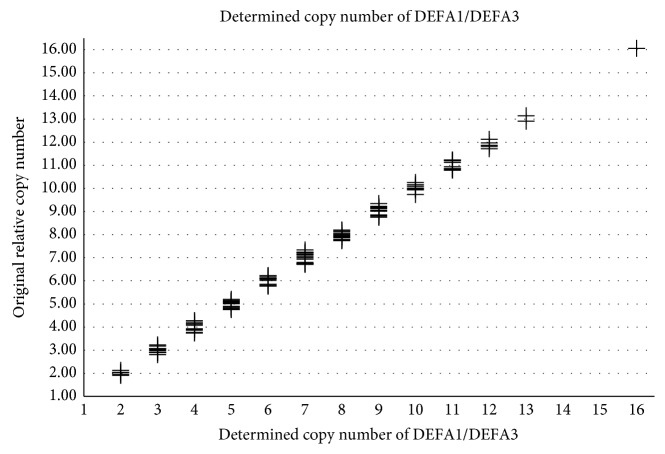
The determined copy number and its original relative copy number of DEFA1/DEFA3. The *x*-axis referred to the finally determined copy number, and the *y*-axis was the original relative copy number obtained from the qPCR method before rounding. The median CNV of a total of 215 critically ill patients was 7.

A qPCR-based assay was developed to detect DEFA1/DEFA3 genomic DNA copy number using the albumin gene as a reference [26, 27]. CNV was calculated by the △△Ct method, which uses the reference gene copy number to perform linear regression [28]. All qPCR reactions (20 *μ*L) were performed on an iCycler Thermal Cycler instrument (Bio-Rad Laboratories, Hercules, CA), containing 1 × iQ SYBR Green Supermix (Bio-Rad Laboratories) and 250 nM each of the forward and reverse primers (DEFA1/DEFA3 forward primer, *5*′*-CCGTCCTTCCCTCTAGACTTAGC-3*′ and reverse primer: *5*′*-GAGCAGATTGCAGCGGACAT-3*′*;* albumin forward primer: *5*′*-TATCGACGACTCTTTACCCTGTCA-3*′ and reverse primer: *5*′*-CCAAAGTCCACACGGAATGC-3*′). PCR cycling parameters were set as 1 cycle at 95°C for 10 min, 40 cycles at 95°C for 10 s, 60°C for 60 s, and 1 cycle at 99°C for 1 min, followed by a melting curve initiating at 60°C and increasing in 0.05°C increments. DNA analysis was performed in triplicate with starting quantities of 50, 10, and 2 ng/L using five-fold serial dilutions, and CNV was the average of results obtained from the three different DNA concentrations used. Copy number was rounded up or down to the closest integer.  Figure 1 was presented to show the original relative copy number before rounding and its finally determined copy number of DEFA1/DEFA3.

### 2.4. Statistical Analysis

DEFA1/DEFA3 copy number in HAIs and controls was analyzed as a continuous variable [18, 20, 29, 30], and comparison of the medians between the two groups was performed by the Mann–Whitney test. Then, in order to clinically apply, we used the median CNV of all 215 patients as a cutoff and divided it into two categories (CNV < 7 copies and CNV ≥ 7 copies) and performed further comparisons using chi-square test. Also, logistic regression was used to select risk factors and to fit the predictive model on the incidence of HAIs. We used binary logistic regression (forward, LR) with an entry level of 0.05 and an exclusion level of 0.10 to identify predictor variables among clinical covariates and lower DEFA1/DEFA3 copy number (CNV < 7). The receiver operating characteristic (ROC) curve was used to evaluate the efficiency of prediction, and the area under the ROC curve (AUROC) > 0.75 was considered as good predictive ability. All statistical tests were two sided and performed by SPSS 20.0 software. Differences with a *p* value < 0.05 were considered significant.

## 3. Results

### 3.1. Patient Characteristics and Outcomes

**Figure 2:**
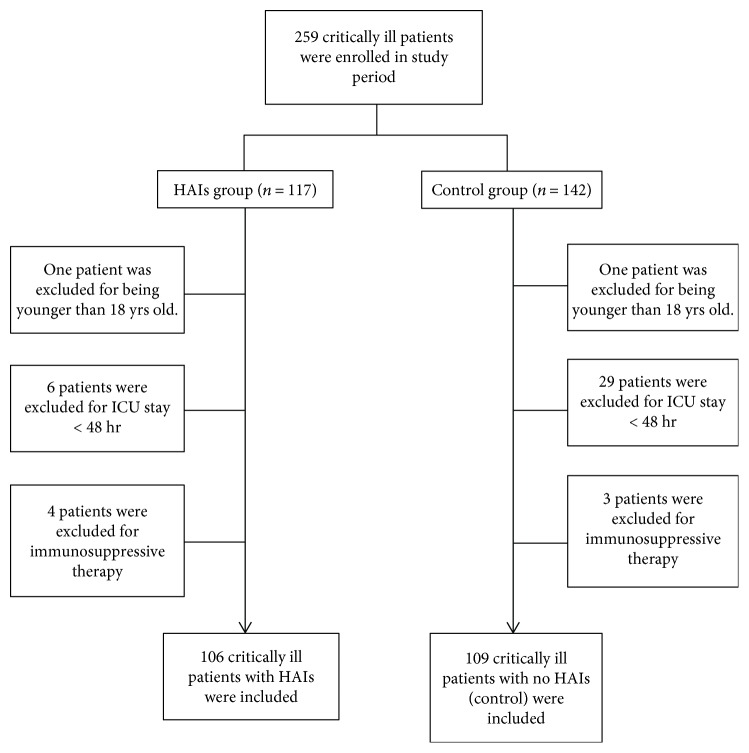
Flow chart of inclusion and exclusion criteria in the HAIs and control groups. From June 1, 2016, to November 30, 2016, 259 critically ill patients were screened for the study, including 117 HAIs patients and 142 controls. In the HAIs group, eleven HAIs patients were excluded from the study due to ICU stay of less than 48 hours (*n* = 6), young age (*n* = 1), or receipt of immunosuppressive therapy (*n* = 4), while 33 controls were excluded for less than 48-hour ICU stay (*n* = 29), young age (*n* = 1), or receipt of immunosuppressive therapy (*n* = 3).

From June 1, 2016, to November 30, 2016, 259 critically ill patients were screened for this study, including 117 HAIs patients and 142 controls. In the HAIs group, eleven HAIs patients were excluded from the study due to ICU stays less than 48 hours (*n* = 6), young age (*n* = 1), or receipt of immunosuppressive therapy (*n* = 4). Thirty-three critically ill patients in the control group were excluded as well. Finally, 215 patients were enrolled in the study including 106 HAIs and 109 controls. Detailed information is shown in Figure 2.

Critically ill HAIs and control patients were not significantly different in terms of age or gender. HAIs patients were more often from the emergency department, general ward, and other ICUs (*p* < 0.05) compared with controls, for which the operating room or recovery room was the main source. Disease severity at ICU admission was indicated by APS, APACHE II, and SOFA scores, which were all significantly higher in the HAIs group (*p* < 0.01). There were dramatic extensions of hospital and ICU stays in patients who developed HAIs (*p* < 0.001). Furthermore, the 28-day mortality in HAIs patients was higher than that in the control group, although these values did not attain statistical significance. Detailed characteristics of each group are shown in Table 1.

### 3.2. Infection Characteristics of HAIs Patients

The infection characteristics of patients with HAIs are listed in Table 2. In critically ill patients who contracted HAIs, the main sources of infection were the respiratory tract (72.6%), abdomen (39.6%), bloodstream (21.7%), and urinary tract (7.5%). Gram-negative bacteria were the most prevalent pathogen in HAIs patients (76.4%), while gram-positive bacteria comprised only 23.6%. Fungi were another type of important infectious pathogen that was present. Multiple infections and multidrug-resistant bacteria (MDRB) were extraordinarily common when critically ill patients contracted HAIs, and among them, MDR *Acinetobacter baumannii* (38.7%) was the most prevalent pathogen.

### 3.3. Distribution of DEFA1/DEFA3 Copy Number

The copy number of DEFA1/DEFA3 ranged from 2 to 16 per diploid genome across all 215 critically ill patients, with a median of 7 copies. In the HAIs group, the copy number of DEFA1/DEFA3 varied from 2 to 12 copies, with a median number of 6. In patients who did not develop HAIs, the genomic copy number of DEFA1/DEFA3 ranged from 2 to 16, with a median of 8. There were significant differences in the distribution of DEFA1/DEFA3 CNV between the two groups (*p* = 0.017). Details of copy number frequencies in the two groups are shown in Table 3.

Additionally, we categorized patients into two groups based on whether their copy number was less than 7 per genome, which was the median of all 215 critically ill patients included in this study. We found that 52.8% of HAIs had fewer than 7 copies compared with only 35.8% in controls. Critically ill patients with lower DFEA1/DEFA3 CNV (CNV < 7) were associated with an increased risk for HAIs (*p* = 0.012, odds ratio (OR), 2.010, 95% confidence interval (CI), 1.164–3.473).

### 3.4. HAIs Prediction Based on DEFA1/DEFA3 CNV

**Figure 3:**
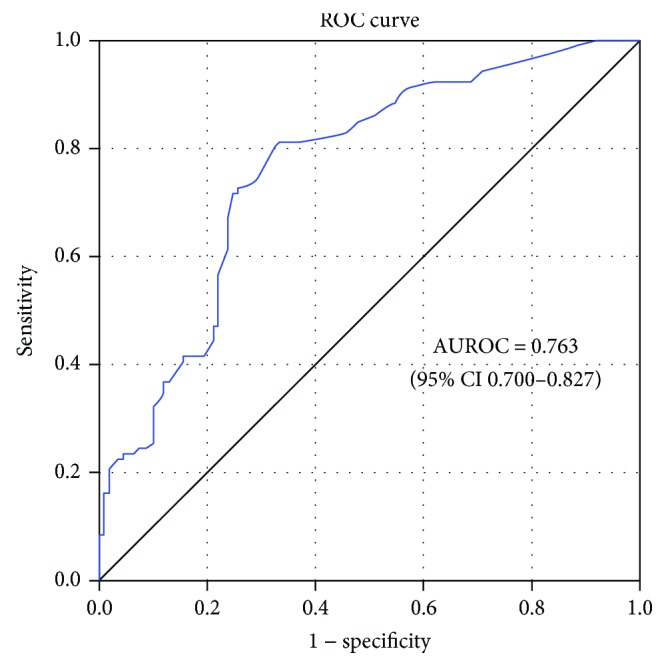
ROC curves of the predictive model and its respective AUROC (95% CIs). The blue curve is the ROC curve for the predictive model. Binary logistic regression was used to fit the model, and the ROC curve was used to evaluate the efficiency of prediction. Area under the ROC curve (AUROC) > 0.75 was considered good predictive ability.

Binary logistic regression was used to select covariates from inherent factors, including sex, age, DEFA1/DEFA3 CNV, and clinical characteristics, including APS score, emergency source, presence of tracheal intubation, and deep venous catheterization within 24 h ICU admission. Ultimately, the multivariate logistic regression model included three covariates, namely CNV < 7, APS score, and emergency source, which were significantly independent predictors for HAIs (Table 4). DEFA1/DEFA3 copy number less than 7 was correlated with higher susceptibility to HAIs (OR 3.014, 95% CI 1.609–5.648), and higher APS score and emergency source were associated with an increased risk for HAIs (APS score: OR 1.142, 95% CI 1.081–1.207; emergency source: OR 2.522, 95% CI 1.269–5.012). We used the ROC curve to measure the predictive ability of our model based on the three covariates. As shown in Figure 3, the model showed good predictive ability with an AUROC of 0.763 (95% CI 0.700–0.827).

## 4. Discussion

Our case-control study demonstrates an association between HAIs and CNV of DEFA1/DEFA3. The distribution of DEFA1/DEFA3 CNV in 215 critically ill patents ranged from 2 to 16 per diploid genome, with a median of 7 copies. DEFA1/DEFA3 copy number was significantly lower in HAIs, varying from 2 to 12 copies with a median of 6, compared with 2 to 16 with a median of 8 in controls (*p* = 0.017). Decreased DEFA1/DEFA3 copy number (CNV < 7) was correlated with an increased risk for HAIs. Lower DEFA1/DEFA3 copy number (CNV < 7) could be used to help predict the incidence of HAIs combined with clinical characteristics (AUROC =0.763, >0.75), indicating the involvement of DEFA1/DEFA3 in the immune response to HAIs.

Consistent with the findings in the present study, DEFA1/DEFA3 has previously been associated with several infectious and inflammatory disease phenotypes. Reduced DEFA1/DEFA3 copy number has been associated with higher susceptibility to recurrent urinary tract infections (UTIs) in children with vesicoureteral reflux [20] and HIV infection [31]. Lower DEFA1/DEFA3 CNV may lead to impaired innate defenses and subdued inflammatory signals, which may ultimately be permissive to infections. Interestingly, higher copy numbers of DEFA1/DEFA3 CNV have been associated with a risk for severe sepsis [18]. This discrepancy may be due to the diverse effects of defensins in different diseases. In sepsis patients, high levels of HNP1–3 may induce excessively activated inflammatory and autoimmune injury that results in the development of severe sepsis [18].

Our study found that lower copy numbers of DEFA1/DEFA3 are associated with a phenotype of a higher risk for HAIs, suggesting a role for HNP1–3 in innate immunity and adaptive immunity in the development of HAIs. This phenotype can be interpreted as a gene dosage effect, where increased copy numbers of DEFA1/DEFA3 would result in elevated levels of HPN1–3 mRNA and protein [32]. HNP1–3 are primarily stored in the azurophil granules of neutrophils and then released into circulation and infection sites in response to infectious pathogens [33]. In addition, HNP1–3 mediate their effects in a dose-dependent manner [33, 34]. Therefore, lower copy numbers of DEFA1/DEFA3 mean lower levels of HNP1–3 in neutrophils or other expression sites, leading to more fragile functioning of antimicrobial activity and immunity. Ultimately, this results in higher risk for HAIs.

Quantitative PCR is an alternative to fluorescence in situ hybridization (FISH), which is the current gold standard to detect CNVs in the genome. Research has shown substantial agreement between qPCR and FISH techniques, indicating qPCR as a viable alternative approach, particularly when starting material is too scarce or cells are too damaged to obtain accurate results from FISH studies [27]. Using quantitative PCR, the copy number of DEFA1/DEFA3 in our study varied from 2 to 16 in critically ill patients, with a median of 7, which is consistent with the finding of the previous studies [17, 18, 20] using the same detection technique. On the other hand, the CNV of DEFA1/DEFA3 varied between 4 and 11 in a sample of 111 control individuals from the UK [15]. This discrepancy may result from the diversities of DEFA1/DEFA3 CNV in different human populations.

Individual differences in HAIs are influenced by hereditary genetic susceptibility of the patients. Previous single nucleotide polymorphism (SNP) studies have shown that over- or underexpression of immunoinflammatory genes such as TNF-*α*, interleukin- (IL-) 1, IL-10, IL-8, interferon gamma, and CD14 receptor is related to infection development in hospitals [35, 36]. We have found that decreased CNV of DEFA1/DEFA3 increases susceptibility to HAIs in critically ill patients, which indicates that genetic variation in innate immunity is a risk factor for HAIs. This finding yields new insight into the pathophysiology of HAIs and may lead to novel potential therapeutic modalities.

Several limitations must be acknowledged in the present study. First, we did not test or verify the mRNA and HNP1–3 expression levels in leukocytes, though several previous studies have found proportional correlations between HNP1–3 levels inside neutrophils and copy numbers of DEFA1/DEFA3 [15, 20]. However, other studies failed to reveal this correlation because HNP1–3 proteins are mainly expressed in bone marrow precursors of neutrophils, promyelocytes, and early myelocytes [11, 18, 33]. Multiallelic CNV in the DEFA1/DEFA3 locus needs to be further investigated. Second, patients enrolled into our cohort were all from the Chinese Han population, but DEFA1/DEFA3 CNV may be different in diverse populations. Thus, the relationship between DEFA/DEFA3 CNV and susceptibility to HAIs in other populations remains to be further elucidated. Finally, though we have established a model to predict the incidence of HAIs, a prospective cohort study is warranted to both validate and improve our model.

## 5. Conclusions

In summary, lower DEFA1/DEFA3 copy number (CNV < 7) is associated with higher susceptibility to HAIs in critically ill patients, indicating that host genetic factors are involved in the development of HAIs. DEFA1/DEFA3 CNV combined with clinical characteristics can predict the incidence of HAIs, which may provide new insights into the pathophysiology of hypersusceptibility to HAIs and lead to new potential therapeutic or prophylactic targets.

## Figures and Tables

**Table 1:** Basic characteristics of two groups at ICU admission.

Characteristics	HAIs **(***n* = 106**)**	Controls (*n* = 109)	*p* value
Age (yr), mean ± SD	61.96 ± 14.24	61.98 ± 13.37	0.992
Male sex, *n* (%)	74 (69.8%)	66 (60.6%)	0.198
Source of ICU admittance, *n* (%)			0.02^∗^
Emergency department	38 (35.8%)	20 (18.3%)	
General ward	9 (8.5%)	7 (6.4%)	
Operating or recovery room	55 (51.9%)	82 (75.2%)	
Other ICU	4 (3.8%)	0 (0%)	
APS score, median, IQR	11 (7–18.25)	6 (5–12)	<0.001^∗^
APACHE II, median, IQR	16 (11–22)	11 (8–16)	<0.001^∗^
SOFA, median, IQR	5 (1–7)	3 (1–6)	0.024^∗^
Length of hospital stay (days), median, IQR	24 (16–34)	16 (11–26)	<0.001^∗^
Length of ICU stay (days), median, IQR	11 (5–17)	4 (2–5)	<0.001^∗^
28-day mortality, *n* (%)	14 (13.2%)	8 (7.4%)	0.181

HAIs: hospital-acquired infections; *n*: number of subjects; APS: Acute Physiology Score, APACHE II: Acute Physiology and Chronic Health Evaluation score II; SOFA: Sequential Organ Failure Assessment score. Values are presented as the mean ± standard deviation (SD), median (interquartile range (IQR)), or *n* (%). Continuous variables with normal distribution are presented as the “mean ± SD”; continuous variables with abnormal distribution are presented as the “median, IQR”; categorical variables are presented as “*n* (%).” Each ^∗^*p* value < 0.05 was considered to be significant. The *p* value was obtained by *t*-test or Mann–Whitney test for continuous variables and chi-square test for categorical variables.

**Table 2:** Infection characteristics of the HAIs group.

Infection characteristics	HAIs (*n* = 106), *n* (%)
Identical infection with positive pathogen culture	101 (95.3%)
Source of infection	
Respiratory	77 (72.6%)
Abdomen	42 (39.6%)
Bloodstream	23 (21.7%)
Urinary tract	8 (7.5%)
Invasive vessel	7 (6.6%)
Wound	5 (4.7%)
More than one locus	45 (42.5%)
Infectious organisms	
Gram-positive bacteria	25 (23.6%)
Gram-negative bacteria	81 (76.4%)
Fungi	43 (40.6%)
Multiple infection^∗^	71 (67.0%)
Multi-drug-resistant bacteria^#^	81 (76.4%)
Specific bacterial infection	
MRSA	1 (0.9%)
ESBL-producing *E. coli*	9 (8.5%)
MDR-*AB*	41 (38.7%)

HAIs: hospital-acquired infections; *n*: number of subjects; MRSA: methicillin-resistant *Staphylococcus aureus*; ESBL: extended-spectrum beta-lactamase; MDR-*AB*: multi-drug-resistant *Acinetobacter baumannii*. Multiple infection^∗^ refers to infection caused by at least two pathogens. Multidrug-resistant bacteria^#^ are defined as isolated pathogens with resistance to at least three types of antimicrobial drugs. A patient might be infected in more than one locus or by more than one organism.

**Table 3:** Distribution of DEFA1/DEFA3 copy number in HAIs patients and controls.

CNV	HAIs (*n* = 106)	Controls (*n* = 109)
2	6	3
3	11	9
4	10	11
5	19	7
6	10	9
7	8	12
8	14	13
9	11	18
10	3	11
11	11	8
12	3	5
13	0	2
14	0	0
15	0	0
16	0	1
Median	6	8
^∗^ *p* value	0.017	

CNV < 7	56 (52.8%)	39 (35.8%)
CNV ≧ 7	50 (47.2%)	70 (64.2%)
^#^OR (95% CI)	2.010 (1.164–3.473)	
^#^ *p* value	0.012	

HAIs: hospital-acquired infections; *n*: number of subjects; CNV: copy number variation. Risk was calculated using odds ratio (OR) (confidence interval (CI)). Each *p* value < 0.05 was considered to be significant. DEFA1/DEFA3 CNV of each group was a continuous variable, and the ^∗^*p* value was obtained by the Mann–Whitney test. CNV was categorized into two groups (CNV < 7 and CNV ≥ 7), and then ^#^*p* value was obtained by chi-square test. The ^#^OR and 95% CI show the risk effect of CNV < 7 to HAIs.

**Table 4:** Logistic regression of DEFA1/DEFA3 copy number and occurrence of HAIs in critically ill patients.

Model	Covariants	*p* value	OR	CI (95%)
Predictive model	CNV < 7^∗^	0.001	3.014	1.609–5.648
	APS	<0.001	1.142	1.081–1.207
	Emergency source^#^	0.008	2.522	1.269–5.012

Risk was calculated by the odds ratio (OR) (confidence interval (CI)). Each *p* value < 0.05 was considered to be significant. APS: Acute Physiology Score; CNV: copy number variation; CNV < 7^∗^: categorical variable, if DEFA1/DEFA3 CNV less than 7 copies = 1, and if CNV ≥ 7, it would be 0; Emergency source^#^: categorical variable and whether the patient was from an emergency department = 1, while if the patient was not from an emergency department = 0. APS scores were continuous variables and entered the regression with the actual value. We use binary logistics regression (forward, LR) to fit the model with an entry level of 0.05 and an exclusion level of 0.10.
